# Piezo‐Acoustic Resistive Switching Behaviors in High‐Performance Organic–Inorganic Hybrid Perovskite Memristors

**DOI:** 10.1002/advs.202308383

**Published:** 2024-01-15

**Authors:** Zehan Liu, Pengpeng Cheng, Ruyan Kang, Jian Zhou, Xiaoshan Wang, Xian Zhao, Jia Zhao, Duo Liu, Zhiyuan Zuo

**Affiliations:** ^1^ Key Laboratory of Laser & Infrared System (Shandong University) Ministry of Education Shandong University Qingdao 266237 P. R. China; ^2^ Center for Optics Research and Engineering Shandong University Qingdao 266237 P. R. China; ^3^ Institute of Novel Semiconductors Shandong University Jinan 250100 P. R. China; ^4^ School of Information Science and Engineering Shandong University Qingdao 266237 P. R. China

**Keywords:** conductive filaments, ion migration, memristors, organic–inorganic halide perovskites, piezo‐acoustic resistive switching

## Abstract

Memristors are regarded as promising candidates for breaking the problems including high off‐chip memory access delays and the hash rate cost of frequent data moving induced by algorithms for data‐intensive applications of existing computational systems. Recently, organic–inorganic halide perovskites (OIHPs) have been recognized as exceptionally favorable materials for memristors due to ease of preparation, excellent electrical conductivity, and structural flexibility. However, research on OIHP‐based memristors focuses on modulating resistive switching (RS) performance through electric fields, resulting in difficulties in moving away from complex external circuits and wire connections. Here, a multilayer memristor has been constructed with eutectic gallium and indium (EGaIn)/ MAPbI_3_/poly(3,4‐ethylenedioxythiophene): poly(4‐styrenesulphonate) (PEDOT: PSS)/indium tin oxide (ITO) structure, which exhibits reproducible and reliable bipolar RS with low SET/RESET voltages, stable endurance, ultrahigh average ON/OFF ratio, and excellent retention. Importantly, based on ion migration activated by sound‐driven piezoelectric effects, the device exhibits a stable acoustic response with an average ON/OFF ratio greater than 10^3^, thus realizing non‐contact, multi‐signal, and far‐field control in RS modulation. This study provides a single‐structure multifunctional memristor as an integrated architecture for sensing, data storage, and computing.

## Graphical abstract

The memristors with EGaIn/MAPbI_3_/PEDOT:PSS/ITO structure are successfully fabricated. The devices present reproducible electrical RS behavior. Importantly, the devices exhibit piezo‐acoustic RS behaviors, enabling non‐contact, multi‐signal, and far‐field control. This phenomenon is strongly dependent on different frequencies and SPL. A physical model is proposed to enhance understanding. This work introduces a single‐structure multifunctional memristor for sensing, data storage, and computing.

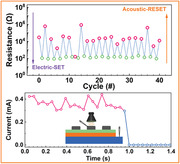

## 1 Introduction

The continuous evolution of a smarter world encompassing neuromorphic computing,^[^ ^1^ ^]^ cloud robotics,^[^ ^2^ ^]^ and the Internet of Things (IoT),^[^ ^3^ ^]^ demands comprehensive, and inevitable innovations of the electronic memories that are currently used on a large scale. However, traditional memory devices, e.g., static random‐access memory (SRAM), dynamic random‐access memory (DRAM), and flash, are approaching their physical limits when scaled down due to enhanced quantum tunnel‐induced charge leakage at the 10‐nanometer scale. Consequently, it is difficult to shrink their sizes to increase storage density storage in a bit‐cost scalable manner. Moreover, modifying the MOS (Metal‐Oxide‐Semiconductor) technology as well as optimizing memory operation to overcome these challenges and achieve further upgrades are also quite difficult.^[^ ^4^ ^]^ As a solution to challenges posed by new non‐volatile memory (NVM) storage devices, Resistive Random‐access Memory (RRAM) has garnered attention for meeting the demands of efficiently handling massive data. Due to its rapid switching speed, ultra‐large‐scale‐integration (ULSI) densities, simple structure, and low power consumption, memory technology experts believe that RRAM based on the memristors is the optimal choice in the mass memory market. This selection aims to streamline production costs by focusing on a select few technologies.^[^ ^5^ ^]^

Memristors are two‐terminal devices with an electrode/active material/electrode capacitor‐like structure. They have received enormous attention for data storage applications due to their advantages in simplistic structure, high integration density, fast switching speed, low power consumption, and high density.^[^ ^6^, ^7^ ^]^ The SET (switching the device from high resistance state to low resistance state, LRS) and RESET (switching the device from low resistance state to high resistance state, HRS) processes correspond to the information storage operation of write and erase, respectively, and can be obtained by the external stimulation with varying voltage amplitude, bias polarities, and other stimuli. The formation and rupture of conductive filaments (CFs) is a common form of resistance change.^[^ ^8^, ^9^ ^]^ To date, a variety of materials have been employed to construct the active material including polymers,^[^ ^10^ ^]^ sulfide,^[^ ^11^ ^]^ graphene oxide,^[^ ^12^ ^]^ and chalcogenide.^[^ ^13^ ^]^ Devices based on these materials have made full progress in the field of RRAM and artificial synapses.^[^ ^14^, ^15^ ^]^ However, most of them have complex structures and cumbersome preparation processes, causing problems such as increased manufacturing costs, decreased production efficiency, and decreased production yields.

Recent research indicates that the development of halide perovskites (HPs) memristors holds the potential to improve current semiconductor process integration and lead to a reduction in operating power consumption. As the core unit to obtain high‐performance memristors, the HPs are commonly the compound with the stoichiometric formula ABX_3_ (A:, e.g., formamidinium (FA^+^), methylammonium (MA^+^), Cs^+^, B:, e.g., Pb^2+^, Sn^2+^; X:, e.g., Cl^−^, Br^−^, I^−^), which assembles in a lattice with the coordination numbers 12 for A, 6 for B, and 8 for X.^[^ ^16^ ^]^ Due to unique crystal structure of organic–inorganic HPs (OIHPs), they have drawn tremendous attention for excellent optical absorption, low exciton binding energy, long electron‐hole diffusion, high defect tolerance, and structural and compositional flexibilities. Given these merits, OIHPs have been widely employed in photodetectors, light‐emitting diodes, solar cells (SCs), and so on.^[^ ^17^, ^18^ ^]^ Although OIHPs SCs displayed superior optical and electrical properties to traditional semiconducting materials, the development is somewhat hindered by the hysteresis during *I–V* scans. It is speculated that this phenomenon should be closely related to ferroelectricity and ion migration, providing the basis for the realization of memristors.^[^ ^19^ ^]^ As a prominent member of OIHPs, MAPbI_3_ has been observed to exhibit a piezoelectric response at room temperature in theoretical calculations and experiments. The asymmetry of MA^+^ would result in a pseudo‐cubic structure rather than an inversely symmetric one.^[^ ^20^, ^21^ ^]^ Therefore, the absence of an inversion center makes self‐polarization particularly pronounced. Consequently, with the 4 mm point group and I4/mcm space group for the MAPbI_3_ at room temperature, the measured effective piezoelectric coefficient d_33_ can exceed 5 pm V^−1^, significantly increasing to 25 pm V^−1^ under illumination due to larger light‐induced dipole moments of MA^+^.^[^ ^22^, ^23^ ^]^ Combining an excellent piezoelectric effect with high tolerance to substrates, OIHPs emerge as a focal point for potential applications in acoustic sensors and transducers. Furthermore, they offer new insights into piezoelectric modulation of ion migration in MAPbI_3_ film. Moreover, owing to its ion mobility phenomenon, OIHPs exhibit resistive switching (RS), making them highly attractive for emerging RRAM.^[^ ^24^ ^]^ Building on this foundation, the modulation of ion migration through other physical fields to bring about changes in the resistance state facilitates achieving non‐contact, far‐field RS modulation. Novel memristors based on piezo‐acoustic RS effects can be obtained based on accurate control and optimization.

As we all know, the OIHPs memristors solve the problems of existing computational systems based on Von Neumann architecture such as high off‐chip memory access delays and inefficient algorithms for data‐intensive applications.^[^ ^25^ ^]^ The OIHPs memristor can avoid the processes of module sampling and transmission to the processing unit after acquiring external information. Previously, various electric field‐driven OIHPs memristors were gradually applied in the fields of multilevel storage, neural computing, hardware security, and so on.^[^ ^26^ ^]^ Electric‐field‐driven OIHPs memristors have difficulties in moving away from complex external circuits and wire connections. By utilizing the excellent photonic response, optogenetics‐inspired tunable synaptic functions, photo‐induced logic gate devices, and artificial retina systems have been achieved by the perovskite films.^[^ ^27^ ^]^ The potential drawbacks of optical control in OIHPs memristors include complex equipment requirements, such as lasers, optical lenses, and photodetectors, sensitivity to ambient light and temperature, relatively high energy consumption, and a need for precise alignment. These factors may limit the applicability of optical control and increase the complexity of the system. Moreover, magnetic fields also provide an opportunity to control perovskites‐based memristors in a remote way and environmentally robust devices capable of operating at high temperatures have been prepared. The magnetic field may be influenced by surrounding elements such as metals and other magnetic materials in the environment, introducing potential instability in certain practical applications. Compared to other field‐modulated memristors, acoustic memristors can operate in complex environments with significantly simplified circuits, showcasing superior adaptability. By adjusting acoustic parameters, memristor control can be achieved without the need for high‐energy devices. This will also greatly improve the efficiency of the system and complete the construction of an integrated architecture of sensing, storage, and computing.

In this work, we systematically studied the piezo‐acoustic RS effect based on the MAPbI_3_ memristors under ambient conditions. The single‐structure multifunctional memristors with eutectic gallium and indium (EGaIn)/MAPbI_3_/poly(3,4‐ethylenedioxythiophene): poly(4‐styrenesulphonate) (PEDOT:PSS)/indium tin oxide (ITO) sandwich‐like structure have been successfully fabricated, where the MAPbI_3_ RS layer was synthesized by a low‐temperature all‐solution process. The devices exhibited reproducible bipolar RS behavior with low SET/RESET voltage (+0.56 V/−0.87 V), stable endurance (5 × 10^3^ cycles), much higher electrical or acoustic ON/OFF ratio (10^4^/10^3^), and excellent data retention property (5 × 10^4^ s), revealing prominent RS characteristics along with stability in air. Based on our principle design and experimental verifications, the devices possessed reproducible acoustic‐HRS which is speculated to be due to the piezo‐acoustic‐RS effect of MAPbI_3_ through the sound waves. A physical model was proposed to inspire the understanding and applications of the piezo‐acoustic‐RS behavior. Based on the multi‐signal, and far‐field control in RS modulation, the MAPbI_3_‐based memristors could be a promising candidate for the future construction of an integrated architecture of sensing, storage, and computing.

## 2 Results and Discussion

**Figure 1:**
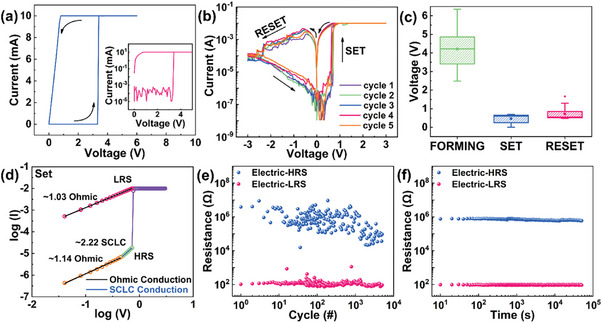
RS properties of the memristors. a) Typical FORMING process. The inset shows the re‐plotted FORMING process of the logarithmic scale of Y. b) Series *I–V* behaviors of the device. c) statistics of the forming, set, and reset voltage distributions depicted as a box‐whisker plot for 50 consecutive cycles. d) Fitted logarithmic *I–V* behavior of positive voltage sweep. e) Endurance test for 5 × 10^3^ cycles. f) Retention test for 5 × 10^4^ s.

For details about the schematic drawing of the memristors, the atomic force microscopy () images, the scanning electron microscopy (SEM) images, the X‐ray diffraction (XRD) patterns, the Raman spectra, the optical absorption spectra, and the photoluminescence (PL) spectra of the samples, see Figures S1–S7 (Supporting Information). The selection of EGaIn material as the top electrode offered the advantage of requiring no additional annealing processing, thereby avoiding significant mechanical or thermal damage to the RS layer. The adaptive deformation capability of the EGaIn electrode enabled the establishment of a stable and tight electrode‐perovskite interface. This adaptability not only helped maintain the integrity of the RS layer but also enhances the reliability under different operating conditions. Due to its low‐temperature solution processability, excellent flexibility, high stability, and remarkable film‐forming ability, the PEDOT:PSS emerged as an ideal choice for crafting uniform and crystalline MAPbI_3_ RS layers. Then, the prepared MAPbI_3_‐based memristors were conducted systematical electrical characterizations. Generally, the FORMING process was necessary to initiate the memory cells to achieve consistent RS behaviors (**Figure** **1**a). A high voltage was employed to induce the generation of vacancy defects and to control the distribution of vacancies for subsequent processes. A positive cyclic sweep (0 V→+6 V→0 V) was applied to complete the FORMING operation and the switching from initial resistance state (IRS, 50.21 MΩ) to LRS (84.60 Ω) occurred at a voltage of ≈+3.32 V (forming voltage), named V_forming_. It has been proved that the RS behavior of a MAPbI_3_‐based memristor is purely associated with the formation and rupture of the CFs rather than the changes in interface valence.^[^ ^28^ ^]^ Afterward, the *I–V* characteristics were collected by applying DC voltage sweeps with a forward SET stop voltage of +3 V and a reverse RESET stop voltage of −3 V. As shown in Figure 1b, the *I–V* curves, illustrated in semilogarithmic scale, exhibited excellent bipolar RS behaviors under a compliance current (I_CC_) of 10 mA. A positive voltage sweeping from 0 to +3 V applied to the top electrode resulted in a sharp current increase and the device was switched from the HRS to the LRS, referred to as the “writing” or SET process. With the voltage scan from +3 to 0 V, the resistance value tended to be constant, proving the non‐volatile feature. For voltage sweep from 0 to −3 V, the resistance slowly increased to the HRS, termed as “erasing” or RESET process. Subsequently, no significant fluctuation had been observed in the switching voltages and resistance for five cycles, suggesting a high reproducibility. RS power consumption of SET process (36.9 mW) and RESET process (1.9 mW) was obtained from *I–V* curves. Additionally, to confirm the uniformity of the memristors, the *I–V* measurements were carried out at five randomly selected areas on the sample. Despite that the RESET voltages and ON/OFF ratios were slightly different, all the devices maintained stable RS operations (Figure S8, Supporting Information). To investigate the switching speed, transient measurements were performed, as shown in Figure S9 (Supporting Information). The tests confirmed that the device has a switching speed of ≈340 ns, much faster than flash memory devices (order of µs). The memristors are competitive with the conventional flash memory and NVMs in switching speed.^[^ ^29^ ^]^

The switching voltages had been collected to investigate the statistics characterizations of the RS performance. The memristors can maintain the switching voltages for more than 50 consecutive cycles (Figure 1c). The average values of the V_forming_, V_set_, and V_reset_ were +3.32, −0.56, −and 0.87 V, respectively, superior to most memory devices.^[^ ^30^ ^]^ Low switching voltage, originating from the low activation energy (E_a_) of iodine vacancies (V_I_s) in the CFs‐dominated memristors, was a primary parameter for the potential applications in low energy‐consuming devices. The mechanism was systematically discussed in the following section.

Basically, the data was stored using two/more very distinct resistance levels assumed by the reversible soft breakdowns. The completion of RS behavior in MAPbI_3_‐based memristors was determined by states of CFs which can be modified by ion migration. Previous research revealed that the MAPbI_3_ layer contained defects, such as vacancies, interstitials, cation substitutions, and anti‐site substitutions, that work as charge‐trapping centers.^[^ ^31^, ^32^ ^]^ Among them, V_I_s were the most active ones in MAPbI_3_ due to their relatively low level of E_a_ (≈0.08 eV). Usually, the ion migration rate (*k*) in solids is estimated from the Arrhenius Equation 1.^[^ ^33^ ^]^

(1) $$k=Ae^{-E_{a}/K_{B}T}$$

where *A* is the pre‐exponential factor, *E_a_* is the activation energy, *K_B_* is the Boltzmann constant, and *T* is the absolute temperature, respectively. Due to the high E_a_ of V_Pb_s (0.80 eV) and V_MA_s (0.46 eV), their migration rates were 1.2 × 10^0^ and 6.5 × 10^5^ s^−1^, much lower than the migration rate of V_I_s (1.7 × 10^12^ s^−1^).^[^ ^34^ ^]^ Therefore, the lowest E_a_ of V_I_s corresponded to the highest migrating rate. Under external stimulations, migrations of V_I_s were so readily that affected the transportation of electrons quite significantly in the close‐packed structure, which generated conductive paths by positively‐charged V_I_s. The FORMING and SET processes were often accompanied by migrations of V_I_s under an electric field. During the forward sweep, the iodide ions hopped toward the top electrode while V_I_s moved to the bottom electrode and formed the CFs gradually. Once the conductive paths of electrons formed, electrons transported along with the CFs and resulted in a significant resistance decrease and converted the device to LRS. Since the SET process occurs on pre‐formed CFs, it was possible to obtain a lower V_set_ (≈0.56 V) than V_forming_ (≈3.32 V).^[^ ^35^ ^]^ On the contrary, I^−^s can also migrate back to the perovskite interior under the reverse voltage sweep during the RESET process, which could recombine with V_I_s and accelerate the annihilation process. As a result, the CFs were gradually dissolved by the ion migration and the current gradually decreased along with the disappearance of the conductive paths. According to the previous reports,^[^ ^36^ ^]^ self‐limited behaviors might be responsible for the fluctuation of resistance. That is to say, the operation variability of the device was generally caused by random growth and dissolution of CFs. However, this phenomenon during the RESET process mentioned above may be caused by a partial rupture of the multiple CFs.^[^ ^37^ ^]^

The *I–V* characteristics of the devices under positive voltage sweeps were drawn in the logarithmic form to interpret the switching mechanism, as depicted in Figure 1d. For the HRS, the ohmic and space charge limited current (SCLC) conduction mechanisms dominated before the switching. At a low voltage bias (0−0.46 V), the slope of 1.03 was indicative of a linear ohmic conduction region (*I*∝*V*), where the number of free carriers generated by thermal fluctuation exceeds the injected carriers. In the high bias region (0.46−0.73 V), all the traps were filled, and the injected carriers dominated over thermally generated ones, corresponding to the SCLC conduction. This classical trap‐controlled SCLC relationship (*I*∝*V* ^2^) can be described by Equation 2.^[^ ^38^ ^]^

(2) $$J=\frac{9}{8}n\varepsilon \mu \left(\frac{V^{2}}{d^{3}}\right)$$

where *J*, *n*, ε, μ, *V*, and *d*are the current density, free carrier concentration, dielectric constant, electronic mobility, biased voltage, and thickness, respectively. Furthermore, inside the perovskite layer, SCLC was a current mechanism closely related to the traps consisting of vacancies, especially V_I_s of the lowest *E_a_*.^[^ ^39^ ^]^ Subsequently, the current increased dramatically by switching HRS to LRS at 0.73 V. In contrast, ohmic behavior consistently dominated in LRS that consistent with the CFs formation as RS mechanism.^[^ ^40^ ^]^ As shown in Figure S10 (Supporting Information), the data in the RESET process exhibited a similar mechanism to the SET process.

To verify the reproducibility of the MAPbI_3_‐based memristors, the cycling endurance measurement was carried out under a read voltage (−0.1 V, 50 ms). As shown in Figure 1e, the endurance property was obtained by applying the continuous SET/RESET stop voltage pulse of +3.0 V/−3.0 V, 50 ms. We have included statistical measures such as mean, standard deviation (SD), and coefficient of variation (CV) of endurance data in Table S1 (Supporting Information). Additionally, we have incorporated a cumulative probability plot of endurance data. As shown in Figure S11 (Supporting Information), uniform switching with low variability in the narrow distribution and well‐separated HRS and LRS were obtained in the endurance. Under the ambient conditions, the memristors can operate for 5 × 10^3^ cycles with fairly stable resistance states during the switching processes. For the beginning 1.3 × 10^3^ cycles, the HRS had a window between ≈100 and ≈10 MΩ achieving a staggering average ON/OFF ratio of ≈10^4^. Nonetheless, in subsequent cycles, the HRS window dropped to between ≈20 and ≈500 KΩ, which resulted in a decrease in the ON/OFF ratio. The drop of the HRS window (cycling‐induced degradation) might be triggered by the excessive accumulation of V_I_s.^[^ ^41^ ^]^ The concentration of local vacancies had a significant impact on diminishing HRS compared to the intrinsic state as they always promote the reduction of *E*_a_ and the formation of partial rupture of the multiple CFs (the augmentation in residual CFs).^[^ ^42^ ^]^ Figure 1f showed the retention distributions (readout voltage: −0.1 V, 50 ms) of the devices with a time of ≈5 × 10^4^ s. Note that the retention level was kept at the same level without obvious deviation and the devices maintained a decent ON/OFF ratio of over 1.5 × 10^4^.

**Figure 2:**
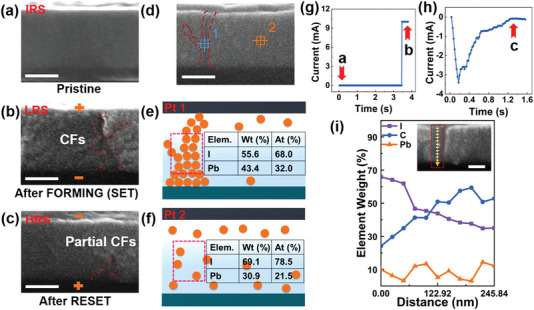
SEM and EDS analysis of the formation and rupture of CFs in MAPbI_3_ perovskite layer. a) The pristine state, corresponding to IRS. scale bar, 100 nm. b) The state after FORMING (SET) process, corresponding to LRS. scale bar, 100 nm. c) The state after the RESET process, corresponding to HRS. scale bar, 100 nm. d) The cross‐sectional SEM image containing the CFs region and non‐CFs region. Point 1: CFs. Point 2: non‐CFs. e) Schematic illustration of the CFs morphology in Point 1. The inset table shows the weight and atomic percentage of the I and Pb elements in Point 1. f) Schematic illustration of the non‐CFs morphology in Point 2. The inset table shows the weight and atomic percentage of the I and Pb elements in Point 2. g) Corresponding *I–t* curve during the forward voltage sweep that led to the image in a and b. (h) Corresponding *I–t* curve during the reverse voltage sweep that led to the image in c. i) Line profile of the EDS intensity of I, C, and Pb elements along the yellow line in the inset from top to bottom. Inset: CFs region of the corresponding line profile of the EDS.

In addition to the previous evidence of the RS mechanism, direct observation of V_I_s CFs during the switching operation was also conducted by cross‐sectional SEM. **Figure** **2**a showed the SEM image of an as‐fabricated device where the MAPbI_3_ layer with a thickness of ≈170 nm, corresponding to the region a in *I–t* curve (Figure 2g). CFs assembled by V_I_s were formed in response to the forward sweep (0 V→+6 V→0 V), and unimpeded electron channels led the memristors to enter LRS state in Figure 2b. This was in accord with the features of CFs observed at region b in Figure 2g. The shape of the CFs resembled that of lightning, and it was also noticed that the diameter of the filament close to the bottom electrode side (∼44.5 nm) was wider than that close to the top electrode side (∼26.7 nm). The morphology of the CFs is also consistent with other research.^[^ ^43^ ^]^ When the negative bias had been applied to the top electrode, the recombination of V_I_s with I^−^s was promoted (region c in Figure 2h). Despite the apparent rupture of the CFs, the residual vacancies still formed partial CFs (Figure 2c). According to Figure 2h, the current dropped gradually and we assumed that the CFs broke from the top to bottom. Eventually, there would be residual CFs retained.^[^ ^44^ ^]^ Moreover, due to the partially formed CFs, the resistance value of HRS was generally not as high as that of IRS, which also corresponded to our experimental results. It can be demonstrated that the devices achieved RS based on the formation and breaking of CFs.

Understanding the RS behavior is critical to device optimization, and in particular, whether I^−^s migration is involved. Energy dispersive X‐ray spectroscopy (EDS) measurement provided the necessary elemental composition information. Considering the conclusion that the Pb element was difficult to migrate while the I element was easy to migrate, we selected point 1 and point 2 for EDS (Figure 2d).^[^ ^45^ ^]^ A schematic illustration of the CFs region (Figure 2e) and non‐CFs region (Figure 2f) was provided to clarify the RS dynamic processes. The specific EDS collection positions were displayed in the dotted red box. Interestingly, EDS results of Points 1 and 2 showed a clear distinction of the weight and atomic percentage of the I and Pb elements. As illustrated in the inset tables of Figure 2e,f, the weight and atomic percentages of I^−^s in Point 1 are 56.6% and 68.0%, respectively, compared with 69.1% and 78.5% in Point 2. These differences revealed that the CFs region formed by the excess V_I_s existed in the film.^[^ ^46^ ^]^ Subsequently, we conducted an EDS line‐scan across the CFs region. It was also observed that the EDS line‐scan results revealed a decrease in  I^−^s content with increasing distance from the electrode. From the 1st measurement point to the 11th, the weight percentage of the I−^−^s decreased by 30.9% unexpectedly. Additionally, the I^−^s at the 10th measurement point site was 34.8%, compared with 35.0% at the 11th measurement point. This difference can be attributed to the fact that there may be fewer defects in the preparation process near the 10th site, which will reduce the ion mobility near this point and lead to the reduction of V_I_s concentration.^[^ ^47^ ^]^ Here we reported the direct observation of V_I_s CFs via SEM imaging combined with compositional EDS analysis of the nanoscale CFs, which provided critical insight into the complex RS dynamic mechanisms.

**Figure 3:**
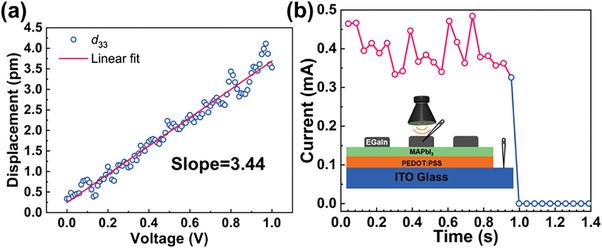
Measurement of piezoelectric coefficient and piezo‐acoustic‐RS. a) The effective piezoelectric coefficient measurement results. b) Reduction of device current stimulated by sound waves of the EGaIn/MAPbI_3_/PEDOT:PSS/ITO memristor. Inset shows the structure used to measure piezo‐acoustic‐RS.

According to previous reports, the displacement of positive and negative electric charge will lead to an electric polarization inside the MAPbI_3_ under pressure and produce the electric charge by the deformation.^[^ ^48^, ^49^ ^]^ In the pursuit of a better understanding of piezoelectric properties, effective longitudinal piezoelectric coefficients (d_33_) were extracted from the V_ac_‐dependent piezo‐responses measurements. As shown in **Figure** **3**a, the d_33_ of the MAPbI_3_ films was ≈3.44 pm V^−1^. The spontaneous polarization of MAPbI_3_ may originate from the orientational polarization of A^+^ dipole, ionic polarization induced by displacements of the positive charge center of MA^+^ relative to the negative charge center of the PbI_3_ cages, and ionic polarization induced by the off‐center displacement of Pb^2+^ in the PbI_6_ octahedron.^[^ ^50^ ^]^ Fan et al. reported that Pb^2+^ showed only 0.01 Å off‐center motion in the PbI_6_ octahedron, which was quite weak for spontaneous polarization.^[^ ^51^ ^]^ The presence of the polar molecule MA^+^ at the center of the cage, creating directional disorders, was a more important factor that accounts for a major portion of the polarization. Structurally, MA^+^ (C_3v_ point groups) would result in the highly symmetrical pseudo‐cubic lattice, causing MAPbI_3_ to lose its inverse symmetry. This asymmetry of MA^+^ meant that an inversion center was absent, leading to particularly pronounced self‐polarization.^[^ ^52^ ^]^ Subsequently, when the MAPbI_3_ was strained by an external force, the internal asymmetric center was further shifted, leading to the destruction of inverse symmetry. Finally, the positive and negative charges would be equally concentrated on the crystal surface, resulting in the macroscopic generation of a built‐in electric field that influenced ion migration and accumulation.^[^ ^53^ ^]^ Hence, utilizing non‐contact acoustic signals to modulate ion migration became an idea to achieve RS behavior.

The fabricated MAPbI_3_‐based memristors were attached to the front of a sound speaker employed to play the sound waves at various frequencies. As expected, the acoustic waves can regulate the RS behaviors due to the piezoelectric effects. The inset of Figure 3b presented the schematic illustration of the configuration used to measure piezo‐acoustic‐RS under ambient conditions. In Figure 3b, when a sine acoustic wave with a sound pressure level (SPL) of 90 dB was generated at 0.954 s, the current of memristors suddenly increased. It should be noted that this phenomenon was substantially different from other methods proposed to improve MAPbI_3_ memristor performance, such as reducing V_forming_ and V_set_ through external stimulation assistance, which still depended on the bias voltage.^[^ ^54^ ^]^ In contrast, the piezo‐acoustic RS produced an irreversible and permanent HRS independently, which did not need to be combined with voltage at all.

**Figure 4:**
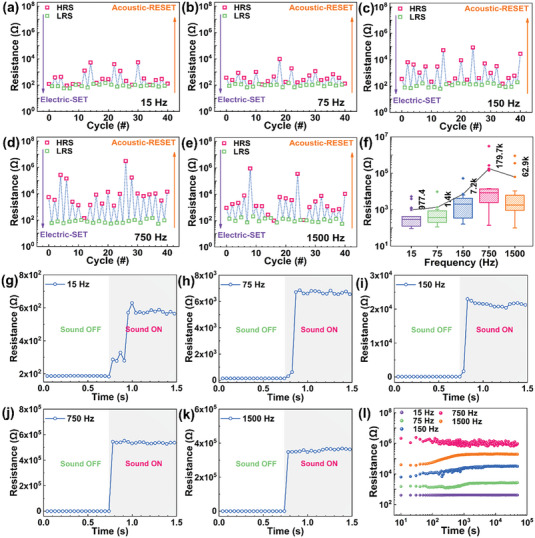
Frequency dependence of the acoustic‐HRSs. a–e) Frequency dependence of the acoustic‐HRSs in successive electric‐SET and acoustic‐RESET cycles. (a) 15 Hz. (b) 75 Hz. (c) 150 Hz. (d) 750 Hz. (e) 1500 Hz. f) Frequency‐dependent statistical distribution of acoustic‐HRSs. (g‐k) Evolution of the device resistance stimulated by different frequency sound waves. g) 15 Hz. h) 75 Hz. i) 150 Hz. j) 750 Hz. k) 1500 Hz. l) Retention test result at different frequencies during 5 × 10^4^ s.

To understand the RS effect, we investigated the frequency responses of the memristors at 15, 75, 150, 750, and 1500 Hz. As shown in **Figure** **4**a,b, the piezo‐acoustic‐RS effect was indistinct, and the RS window was imperceptible with an electric‐SET voltage of +3 V and stimulation of 15 and 75 Hz sine sound waves. Therefore, the small average ON/OFF ratio (≈10^1^) and continuous piezo‐acoustic‐RS failure increased the bit error rate of the storage device. At 150 Hz (Figure 4c), there were still many piezo‐acoustic‐RS failures during the cycles although the average ON/OFF ratio increased to ≈10^2^. In contrast, the phenomenon was well demonstrated under the stimulations of 750 and 1500 Hz signals (Figure 4d,e). The average ON/OFF ratio reached an impressive value of ≈10^3^ under 750 Hz, significantly reducing piezo‐acoustic‐RS failures. To further explore this phenomenon, the statistical distribution of acoustic‐HRSs was investigated in a box‐whisker plot, as shown in Figure 4f. The average value of the HRS can reach an astonishing 179.7 KΩ when stimulated by 750 Hz sound waves, providing a satisfactory window to obtain superior error tolerance.^[^ ^55^ ^]^

Longitudinal sound waves transmitted through the ambient air and caused local compression in the memristors.^[^ ^56^ ^]^ When stimulated by acoustic waves, the memristors were subjected to compressive forces that brought deformations in the sample. In reaction, a piezoelectric potential was created between the two electrodes.^[^ ^57^ ^]^ The polarization charges generated by piezoelectric potential contribute to the migration of I^−^s to balance this potential. Low‐frequency sound waves made it difficult to promote the recombinations of V_I_s and I^−^s and brought about small changes in resistance and even RS failure. Under the external sound pressure, each different film should correspond to different sound frequencies suitable for regulating ion migration. Stronger piezo‐acoustic‐RS will occur when the driving frequency of external forced vibration is close to this frequency. Therefore, the film would vibrate violently, and recombination of excess V_I_s with I^−^s would be maximum, promoting the annihilation process of the V_I_s and enhancing piezo‐acoustic‐RS behavior. Continuous RS behaviors were more pronounced at 750 Hz than that at 1500 Hz, which provided a higher volume of energy density for realizing the rupture of V_I_s CFs.^[^ ^58^ ^]^ The closer the sound frequency is to 750 Hz, the higher the success rate and degree of CFs fractures are. Suitable sound frequencies for regulating ion migration will result in a stable RS effect.^[^ ^59^ ^]^ The *I–V* characteristics of the devices under negative voltage (0 V→−3 V) without the sound, and with the sound at 15, 75150, 750, and 1500 Hz of 90 dB were drawn in the logarithmic form to show the trap‐filled limit voltage (*V_TFL_*), as depicted in Figure S12 (Supporting Information). The current increased from the linear ohmic region through a trap‐filled limit, then eventually to the quadratic Child's region along with the increasing bias voltage. Moreover, *V_TFL_* was calculated the trap density (*n_trap_*) by the Equation 3:^[^ ^60^ ^]^

(3) $$n_{trap}=\frac{2\varepsilon \varepsilon_{0}}{qL^{2}}V_{TFL}$$

where *n_trap_*, *q*, *L*, ε, ε_0_, and *V_TFL_* denoted the trap density, elemental charge, MAPbI_3_ layer thickness, dielectric constant, and vacuum permittivity of perovskite film, trap‐filled limit voltage, respectively. Therefore, *n_trap_* is related positively to *V_TFL_*. The *V_TFL_* of MAPbI_3_‐memristors without the sound and with the sound at 15, 75150, 750, and 1500 Hz of 90 dB were 1.581, 1.499, 1.297, 1.216, 0.932, and 1.054 V, respectively. The MAPbI_3_‐memristor with sound at 750 Hz has a smaller *V_TFL_* than the others, suggesting that the device has fewer defect states, such as the V_I_s.^[^ ^61^ ^]^ This also provided evidence for a reduction in the number of V_I_s caused by sound.

To further investigate the piezo‐acoustic RS behaviors of the memristors and their impact on the switching performances, we studied the piezo‐acoustic‐RS speed at different acoustic frequencies. As illustrated in Figure 4g–k, the sound waves applied to the memristors were 90 dB at 15, 75, 150, 750, and 1500 Hz, respectively. Moreover, with the sampling frequency set at 25 Hz, the piezo‐acoustic‐RS speed was highly dependent on the frequency. At 15, 75, and 150 Hz, the RS speed was ≈0.28, ≈0.12, and ≈0.08 s, respectively (Figure 4g–i).^[^ ^62^ ^]^ After that, the device reached stable HRSs (≈5 × 10^2^, ≈6 × 10^3^, and ≈2 × 10^4^ Ω, respectively). As depicted in Figure 4j,k, the memristor achieved piezo‐acoustic‐RS in less than 0.04 s at 750 and 1500 Hz, heading to achieve stable HRSs. The relationship between the piezo‐acoustic‐RS speed and the sound frequency showed from the side that it was indeed the sound waves that caused the RS behavior. To provide further evidence, piezo‐acoustic‐RS speed under sound waves between 25 and 65 Hz in steps of 10 Hz was studied in Figure S13 (Supporting Information). The piezo‐acoustic‐RS speed at 25, 35, 45, 55, and 65 Hz was 0.28, 0.28, 0.20, 0.20, and 0.12 s, respectively, clearly demonstrating the high‐frequency dependence.

Ion mobility can be suppressed by the strain, and higher frequency acoustic signals may provide a higher probability for vacancy annihilation.^[^ ^63^ ^]^ Therefore, the higher sound frequency in a certain range, the easier it was to promote the CFs rupture. High‐frequency sound waves would squeeze the MAPbI_3_ more vigorously, producing a more effective piezoelectric effect. In this case, the ion migrations were suppressed, and the recombinations of ions and vacancies were accelerated, resulting in a shorter CFs rupture time. Additionally, the E_a_ of V_I_s enhanced by a significant reduction of V_I_s under the compressive strain area, meaningful for the rupture of CFs and the transition to HRS.^[^ ^64^ ^]^ It is obvious to find that the switching speed of non‐volatile piezo‐acoustic‐RS and acoustic‐HRS tend to be affected by frequency variations.^[^ ^65^ ^]^

The retention performances were obtained under the ambient condition to further evaluate the nonvolatile properties of piezo‐acoustic‐RS. Not surprisingly, the acoustic‐HRSs at different frequencies were found to be stable without any detectable degradation over 5 × 10^4^ s with a constant readout voltage of −0.1 V, 50 ms, as schematically illustrated in Figure 4l. The acoustic‐HRSs induced by different frequencies are stable and show a narrower distribution (Figure S14, Supporting Information). The well‐maintained acoustic‐HRSs at different frequencies indicated that the regulation of piezo‐acoustic‐RS to V_I_s CFs was stable, laying a foundation for long‐term data storage. This phenomenon provided potential evidence for the RS behavior dominated by ion migrations under the external stimulus. In contrast to electrical retention, acoustic retention results induced a float in the resistance due to non‐uniformity and complexity CF caused by the vibrational motion of sound waves.

**Figure 5:**
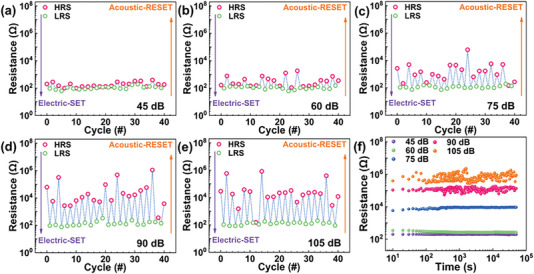
SPL‐dependence of the acoustic‐HRSs and retention property. a–e) SPL‐dependence of the acoustic‐HRSs in successive electric‐SET and acoustic‐RESET cycles. (a) 45 dB. (b) 60 dB. (c) 75 dB. (d) 90 dB. (e) 105 dB. f) Retention test results at different SPL during 5 × 10^4^ s.

In order to investigate SPL dependence of the acoustic‐HRSs, a commercial loudspeaker and an adjustable amplitude were employed as acoustic sources. As depicted in **Figure** **5**a–e, the dual‐dimensional hybrid RS cycle results were obtained by the electric‐SET and acoustic‐RESET to show the SPL dependence. The acoustic‐HRSs at 750 Hz and different SPL (45, 60, 75, 90, and 105 dB, respectively) were investigated under ambient conditions. Under the condition of 45 and 60 dB, the average ON/OFF ratio was ≈1.9 and 4.5 (Figure 5a,b). Ion migrations would not be restricted under low SPL conditions and the CFs formed by electric‐RS did not break.^[^ ^66^, ^67^ ^]^ In this case, HRS simply increased from ≈100 to ≈300 Ω under the influence of sound waves of 45 and 60 dB. As the SPL rose from 60 to 75 dB, the average ON/OFF ratio increased to ≈50 (Figure 5c). It was clear that indestructible CFs under previous SPL conditions ruptured under higher SPL of 45 and 60 dB.^[^ ^68^ ^]^ However, there were still some piezo‐acoustic‐RS failures affecting the operation stability of memristors. Subsequently, similar trends could be observed where the SPL further increased to 90 and 105 dB (Figure 5d,e). The acoustic‐HRSs became uniform during electric‐SET and acoustic‐RESET cycles. The average ON/OFF ratios of 90 and 105 dB at 750 Hz reached ≈10^3^, which is 20 times higher than the result at 75 dB. Moreover, RS exhibited small differences between 90 and 105 dB because they were close to the IRS, where localized vacancy concentration reached an unprecedented minimum, even comparable to the initial concentration. The acoustic‐HRSs hardly increased when the SPL reached 105 dB, which is close to the IRS of the material determined by the intrinsic vacancy concentration. The residual amount of multiple or incomplete rupture CFs would almost disappear under the sound waves with high SPL.^[^ ^69^ ^]^ Figure 5f showed the retention results (read voltage: −0.1 V, 50 ms) at different SPL during 5 × 10^4^ s. The corresponding statistical distribution was shown in Figure S15 (Supporting Information). The reproducible RS with a large ON/OFF ratio was far superior to other pure electric‐driven HPs memristors such as MA_3_Sb_2_Br_9_ and Cs_3_Bi_2_I_9_, indicating ultralow energy consumption.^[^ ^70^, ^71^ ^]^ Generally, this phenomenon proved that the higher SPL stimulation could produce more energetic sound waves for regulating the RS behavior.^[^ ^72^ ^]^ Based on the above analysis, we speculated that piezo‐acoustic mechanical stress affected the migration of ions. Higher frequencies and SPL lead to a more thorough annihilation of V_I_s, resulting in rare residual CFs and ultimately higher HRS. In Table S2 (Supporting Information), we have aggregated the performance parameters of the MAPbI_3_‐based memristors alongside those of other memristors. Notably, our device demonstrated exceptional performance, surpassing or at least comparable to the performance of other memristors in the comparison.

**Figure 6:**
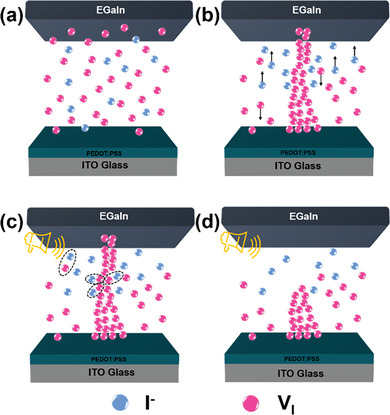
Schematic of the RS mechanism in the EGaIn/MAPbI_3_/PEDOT:PSS/ITO memristor. a) Initial state showing randomly distributed V_I_s. b) CFs construction after FORMING or SET process. c) Recombination of excess V_I_s with I^−^s and annihilation of V_I_s stimulated by sound waves. d) Stable acoustic‐HRSs.

Consequently, we proposed the RS mechanism for the formation and rupture of the CFs based on hybrid electric and piezo‐acoustic RS effects, as illustrated in **Figure** **6**. Initially, a small quantity of V_I_s was randomly dispersed in the MAPbI_3_ films, and the device was in LRS due to the intrinsic high resistance characteristic (Figure 6a). After applying a positive bias to the top electrode in the FORMING process (The subsequent sweep was the SET process), V_I_s with positive charge migrated toward the bottom electrode and accumulated until CFs were constructed (Figure 6b). Subsequently, sound signal stimulation promoted the recombination of excess V_I_s with I^−^s and accelerated the V_I_s annihilation (Figure 6c). Therefore, the V_I_s concentration significantly decreased. Eventually, the CFs ruptured, and the device switched to HRS again (Figure 6d).

## 3 Conclusion

In summary, we reported single‐structure multifunctional memristors with EGaIn/MAPbI_3_/PEDOT:PSS/ITO sandwich‐like structure. Under ambient conditions, the memristors successfully presented robust RS behaviors, including low SET/RESET voltages (+0.56 V/−0.87 V), stable endurance (5 × 10^3^ cycles), ultrahigh electrical and acoustic ON/OFF ratios (10^4^ and 10^3^), and long data retention time (5 × 10^4^ s). Moreover, the RS model, originating from the formation/diffusion of CFs derived from V_I_s migration, was proposed based on direct observations and compositional analysis of the nanoscale conductive regions. The presence of the polar molecule MA^+^ at the center of the cage brought the directional disorder and polarization, accounting for the piezoelectricity of MAPbI_3_ (d_33_ = 3.44 pm V^−1^). Consequently, piezo‐acoustic RS effects can tune the evolution dynamics of V_I_s by inhibiting their formation or accelerating their annihilation, eventually switching the memristor to HRS. The underlying principles related to the frequency and SPL dependences of the piezo‐acoustic‐RS effects were investigated in depth. This work provides an attractive idea for the design and fabrication of an integrated architecture for sensing, data storage, and computing within a single memristor.

## 4 Experimental Section

### Perovskite Synthesis and Device Fabrication

All chemicals in this work were commercially accessible. Lead iodide (PbI_2_, 99.99%), Methylammonium iodide (MAI, >98.00%), PEDOT:PSS, and dimethylsulfoxide (DMSO, AR) were purchased from Shanghai Aladdin Company. All materials were used directly without further purification.

The MAPbI_3_ films were deposited directly on ITO‐coated glass by a one‐step low‐temperature solution processing technology at room temperature under ambient conditions (humidity ≈47%, 24 °C). Before the device fabrication, the commercial ITO glass substrates were ultrasonically cleaned with detergent, DI water, acetone, and ethanol for 20 min, and dried with nitrogen flow. The substrates were treated with oxygen plasma for 2 min to make them more hydrophilic. First, the perovskite precursor solution was formed by adding MAI (1 mmol) and PbI_2_ (1 mmol) to 1 mL of DMSO. Second, the MAI and PbI_2_ were fully dissolved in DMSO through stirring at 70 °C for 10 h. Next, the PEDOT:PSS layer was deposited on the ITO glass substrate with a rate of 3000 revolutions per minute (r.p.m.) for 40 s, and the layer was baked on a hot plate at 70 °C for 5 min. Subsequently, the MAPbI_3_ perovskite precursor solution was spin‐coated at 3000 r.p.m. for 30 s. Then the films were dried in air and heated on a hot plate at 70 °C for 10 min. Finally, EGaIn (75% Ga and 25% In) were utilized as top metal electrodes. The device areas was determined by measuring the circular or elliptical contact area of the EGaIn from the underside using a ruler and a microscope. The majority of the devices exhibited areas of ≈0.04 cm^2^. The PEDOT:PSS thickness was determined to be ≈20 nm.

### Characterization

An atomic force microscopy (Horiba, LabRAM Nano) was used to determine the morphological images of the MAPbI_3_ surface. The scanning electron microscopy (SEM) images were obtained using Hitachi S‐4800 and JSM‐7610F. The X‐Ray diffraction patterns were collected by Rigaku, Smartlab X‐ray diffractometer with Cu Kα radiation (λ = 1.54184 Å). The Raman spectrum of the MAPbI_3_ film was obtained using a T64000 LabRAM confocal Raman instrument (Horiba) equipped with a 532 nm laser. The UV—vis absorption spectra were measured using a METASH V‐5100 spectrophotometer. A spectrograph (Ideaoptics, NOVA 2000) with 375 nm semiconductor laser (PicoQuant, Taiko PDL M1) had been used to receive the steady‐state photoluminescence (PL) signal. *I–V* measurements were carried out by a Keithley 4200A semiconductor parametric analyzer combined with a SUSS PM5 probe station. A commercial programable speaker was used as a sound source for testing the piezo‐acoustic‐RS performance. All measurements were performed at room temperature under ambient conditions.

## Conflict of Interest

The authors declare no conflict of interest.

## Supporting information

Supporting Information

## Data Availability

The data that support the findings of this study are available from the corresponding author upon reasonable request.
