# Supplementary material for: Piezo‐Acoustic Resistive Switching Behaviors in High‐Performance Organic–Inorganic Hybrid Perovskite Memristors
Source: Adv Sci (Weinh). 2024 Jan 15;11(10):2308383. doi: 10.1002/advs.202308383 (PMC10933641; doi:10.1002/advs.202308383)
Supplement: Supplementary file 1 — Supporting Information [file ADVS-11-2308383-s001.pdf]

ADVANCED SCIENCE
Open Access

Supporting Information

for *Adv. Sci.*, DOI 10.1002/advs.202308383

Piezo-Acoustic Resistive Switching Behaviors in High-Performance Organic–Inorganic Hybrid Perovskite Memristors

Zehan Liu, Pengpeng Cheng, Ruyan Kang, Jian Zhou, Xiaoshan Wang, Xian Zhao, Jia Zhao, Duo Liu* and Zhiyuan Zuo**

Supporting Information

Piezo-Acoustic Resistive Switching Behaviors in High-Performance Organic-Inorganic Hybrid Perovskite Memristors

Zehen Liu, Pengpeng Cheng, Ruyan Kang, Jian Zhou, Xiaoshan Wang, Xian Zhao, Jia Zhao, Duo Liu and Zhiyuan Zuo**

Z. Liu, P. Cheng, J. Zhou, X. Wang, Prof. X. Zhao, Prof. J. Zhao, Prof. Z. Zuo
Key Laboratory of Laser & Infrared System (Shandong University), Ministry of Education, Shandong University
Qingdao 266237, P. R. China
E-mail: **zuozhiyuan@sdu.edu.cn**

Z. Liu, P. Cheng, J. Zhou, X. Wang, Prof. X. Zhao, Prof. Z. Zuo
Center for Optics Research and Engineering, Shandong University
Qingdao 266237, P. R. China

Prof. J. Zhao
School of Information Science and Engineering, Shandong University
Qingdao 266237, P. R. China

Prof. D. Liu, Prof. Z. Zuo, R. Kang
Institute of Novel Semiconductors, Shandong University
Jinan 250100, P. R. China
E-mail: **liuduo@sdu.edu.cn**

Section 1. Schematic drawing of the memristors.

A schematic illustration of the MAPbI3-based memristors with a vertically aligned structure was displayed in Figure S1, which showed that the device was composed of the EGaIn top electrode, a $MAPbI_3$ RS layer, a protective layer of PEDOT:PSS, and a bottom electrode of ITO on a glass substrate.

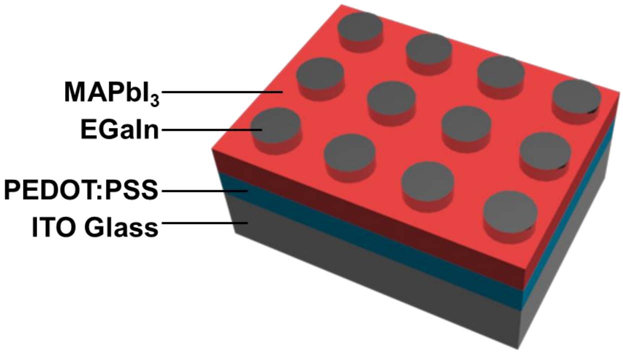

Figure S1. Schematic drawing of the memristors.

Section 2. AFM image of the $MAPbI_3$ film surface.

Figure S2 presented the atomic force microscopy (AFM) topography image of the $MAPbI_3$ film, which showed a relatively uniform surface of the thin film with the root mean squared (RMS) roughness of only 36.56 nm.

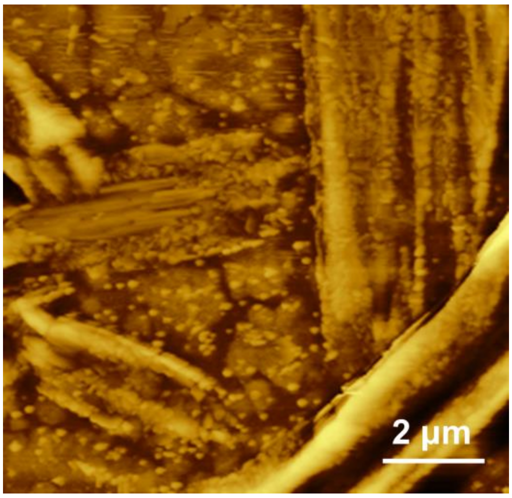

Figure S2. AFM image of the $MAPbI_3$ film surface on a glass substrate.

Section 3. Top-view SEM image of surface morphology

The top-view scanning electron microscopy (SEM) image (Figure S3) confirmed high surface coverage and reveals uniform perovskites film of $MAPbI_3$ surface which was deposited by a one-step solution method under ambient condition.

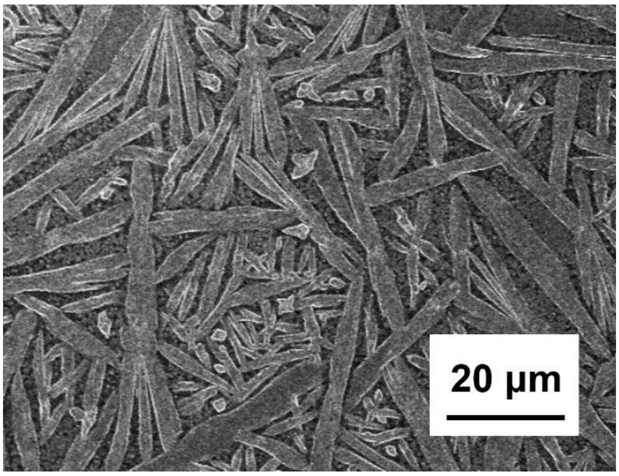

Figure S3. Top-view SEM image of surface morphology of the $MAPbI_3$ film on the PEDOT:PSS/ITO/glass substrate.

Section 4. XRD pattern of the $MAPbI_3$ film

As illustrated in Figure S4**,** an X-ray diffraction (XRD) pattern was performed to analyze the crystallization of synthesized $MAPbI_3$ samples. This clearly exhibited intense diffraction peaks located at 14.15°, 19.98°, 28.47°, 31.95°, and 40.54° that correspond to the (110), (200), (220), (310), and (400) lattice planes of $MAPbI_3$ film, respectively.[1,2]

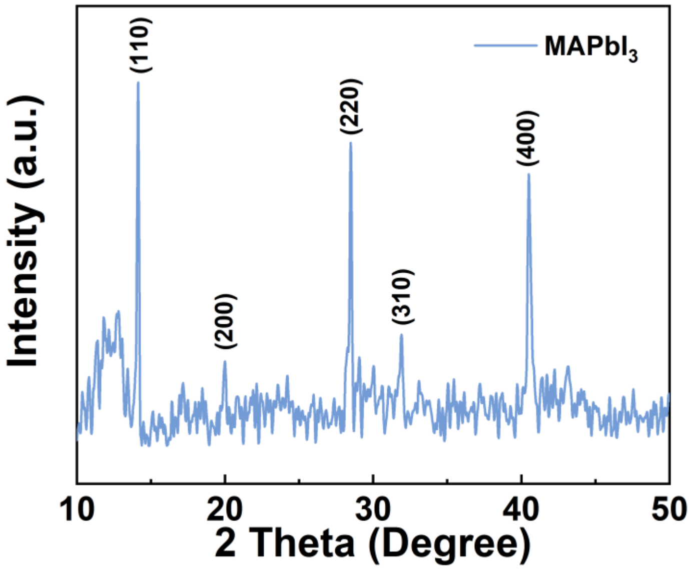

Figure S4. XRD pattern of the $MAPbI_3$ film deposited on a glass substrate.

Section 5. Raman spectroscopy of the $MAPbI_3$ film

In addition, Raman spectroscopy with a range from 50 to 450 cm^{-1} provided a way to selectively study the structural dynamics of MA^+. As illustrated in Figure S5, the modes located around 94 cm^{-1} originated from the bending or stretching motions of the octahedral PbI_6^{4-} framework.[3,4] The experimentally measured Raman vibrations between 120 and 400 cm^{-1} can be attributable to stretching, wagging, and MA−MA asymmetrical stretching modes. Interestingly, the peaks at 120 and 154 cm^{-1} were clear markers of vibrational modes of MA^+, and those at 183, 200, 248, 299, and 398 cm^{-1} were purely associated with the torsional modes of the MA^+.[5,6] Since MA^+ may be disordered around eight possible orientations in the octahedral PbI_6^{4-} framework, there was an intrinsic dipole moment of MA^+.[7] Therefore, the Raman spectrum confirmed that an intrinsic dipole MA^+ existed in our as-fabricated $MAPbI_3$ perovskites, which may induce a structural distortion of the framework and generate crystal structure without inversion symmetry related to ferroelectric properties.

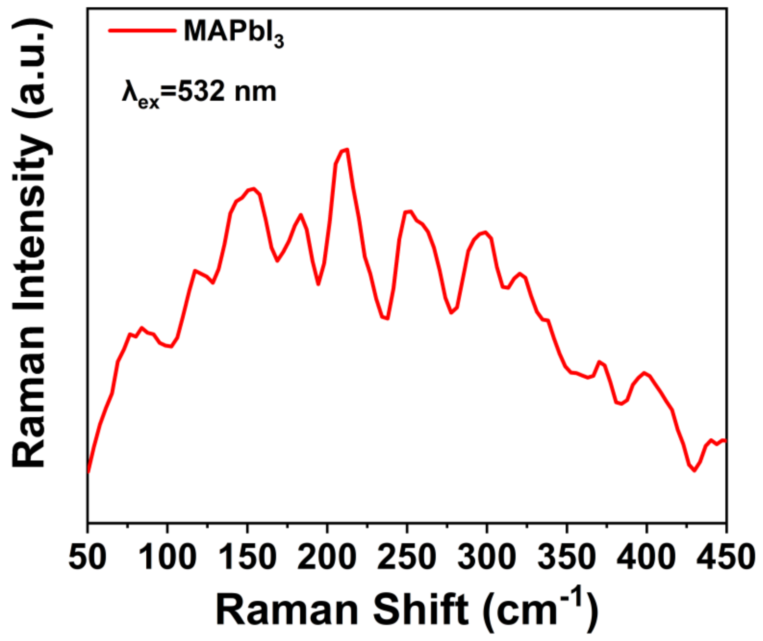

Figure S5. Raman spectroscopy of the $MAPbI_3$ film on a glass substrate.

Section 6. Absorption spectrum of the $MAPbI_3$ film

The absorption spectrum of $MAPbI_3$ showed a broad absorption edge at approximately 765 nm (Figure S6).

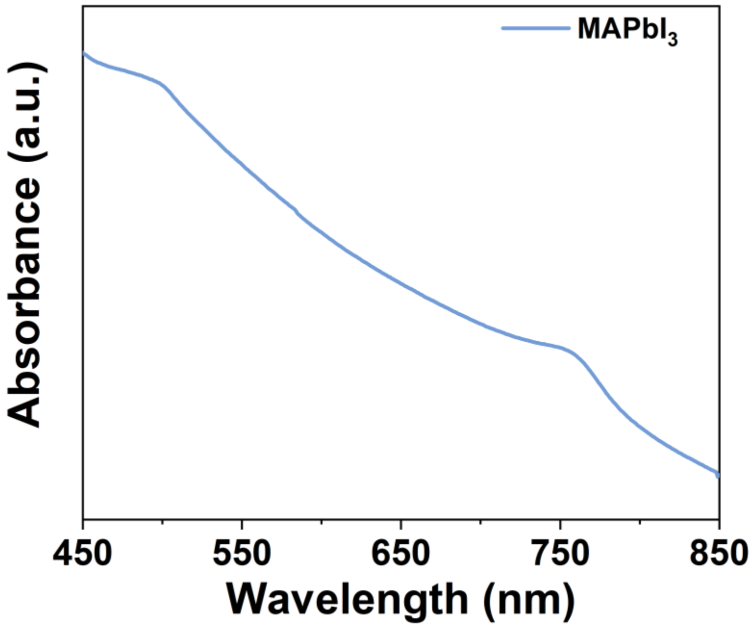

Figure S6. Absorption spectrum of the $MAPbI_3$ film deposited on a glass substrate.

Section 7. The PL spectrum of the $MAPbI_3$ film

Moreover, the normalized photoluminescence (PL) spectrum of $MAPbI_3$ was also measured in Figure S7. A clear PL peak at 771 nm that originated from the interband radiative recombination of photo-induced carriers can be observed with an exciting wavelength of 375 nm.

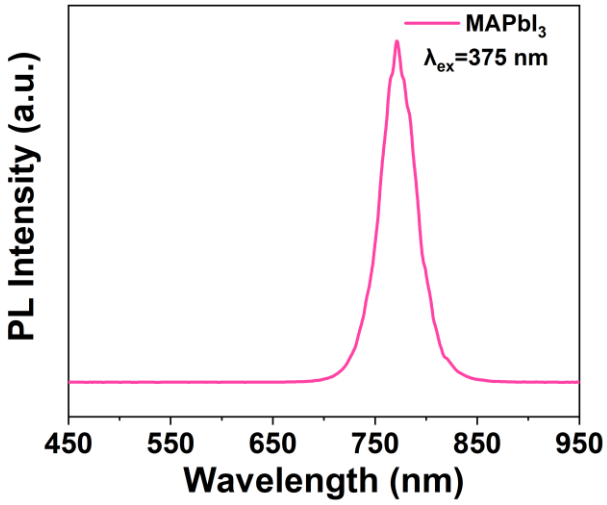

Figure S7. The PL spectrum of the $MAPbI_3$ film deposited on a glass substrate. (excitation λ = 375 nm)

Section 8. Typical *I-V* characteristics at different points.

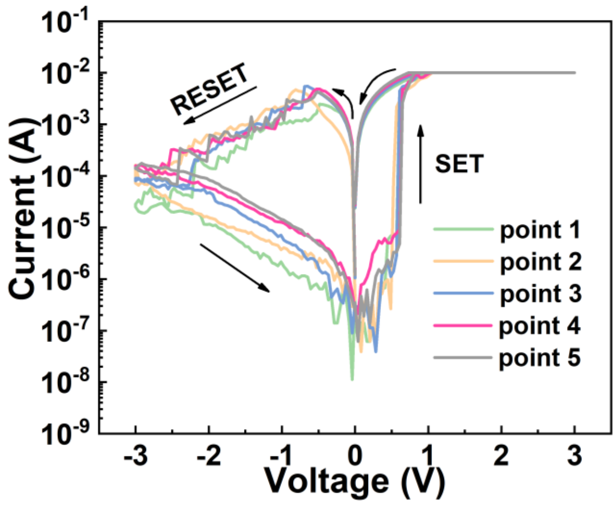

Figure S8. Typical *I-V* characteristics of the EGaIn/$MAPbI_3$/PEDOT:PSS/ITO memristors at different points.

Section 9. The switching speed measurement result.

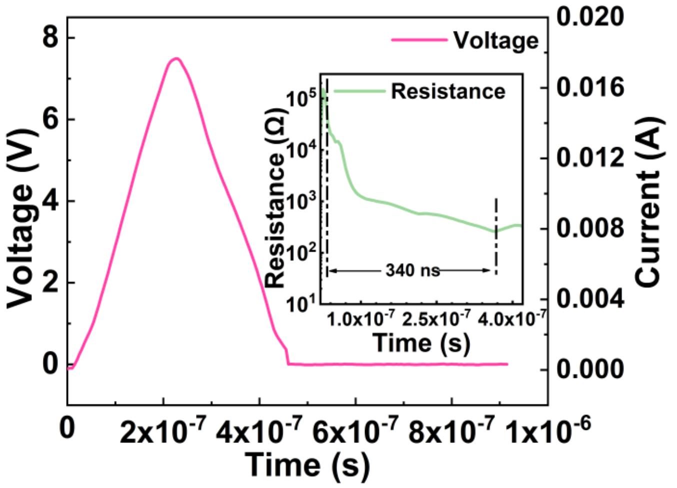

Figure S9. The switching speed measurement result of the EGaIn/$MAPbI_3$/PEDOT:PSS/ITO memristors.

Section 10. Fitted logarithmic *I*-*V* behavior of negative voltage sweep.

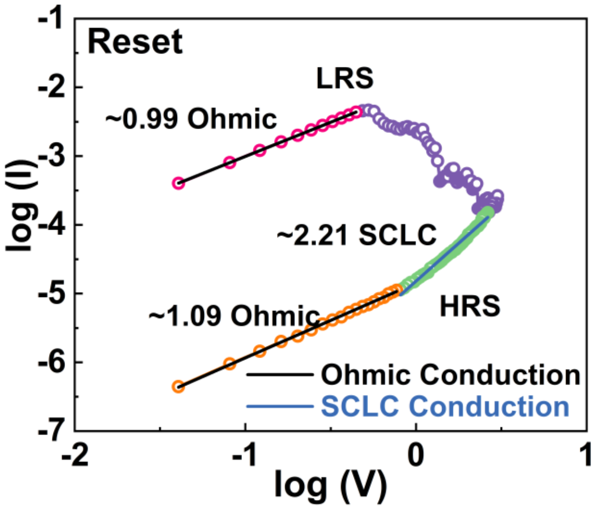

Figure S10. Fitted logarithmic *I*-*V* behavior of negative voltage sweep.

Section 11. Statistical measures of endurance data.

Table S1. Statistical measures of endurance data.

	mean	SD	CV
HRS	1.05 MΩ	1.31 MΩ	125.35%
LRS	123.00 Ω	95.17 Ω	77.38%

Section 12. Cumulative probability plot of electrical endurance data

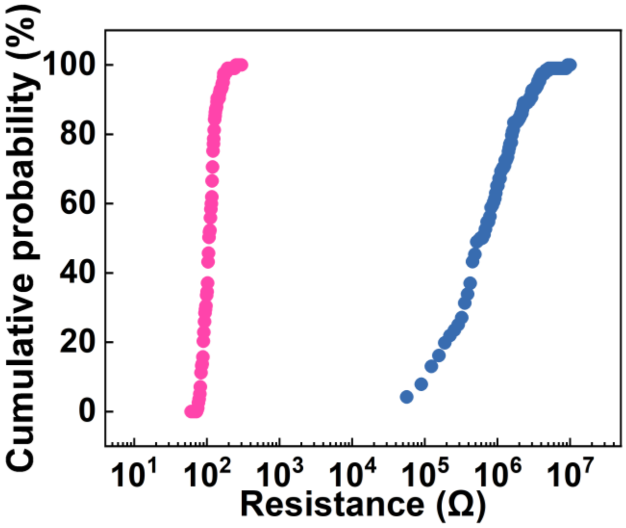

Figure S11. Cumulative probability plot of electrical endurance data.

Section 13. The V_{TFL} of the $MAPbI_3$-memristors without the sound and with the sound at 15, 75,150, 750, and 1500 Hz of 90 dB.

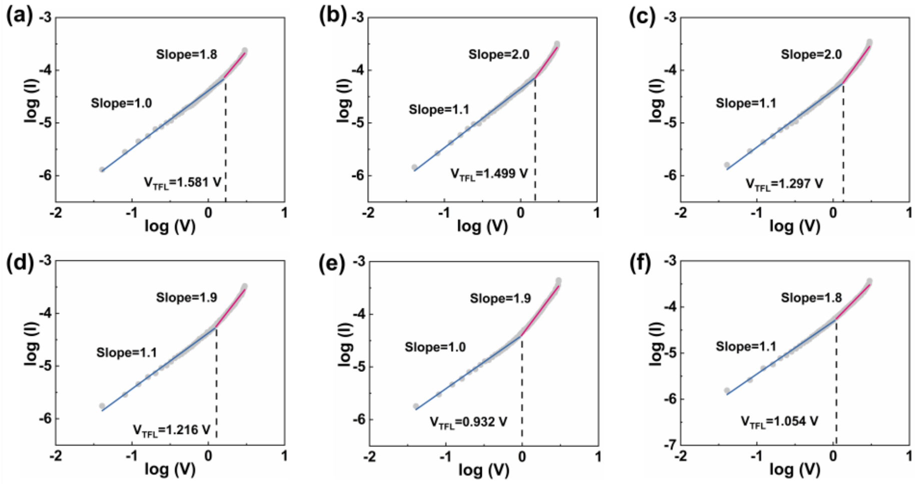

Figure S12. The V_{TFL} of the $MAPbI_3$-memristors without the sound and with the sound at 15, 75,150, 750, and 1500 Hz of 90 dB. (a) without the sound. (b) 15 Hz. (c) 75 Hz. (d) 150 Hz. (e) 750 Hz. (f) 1500 Hz.

Section 14. Evolution of the device resistance stimulated by different frequency sound waves.

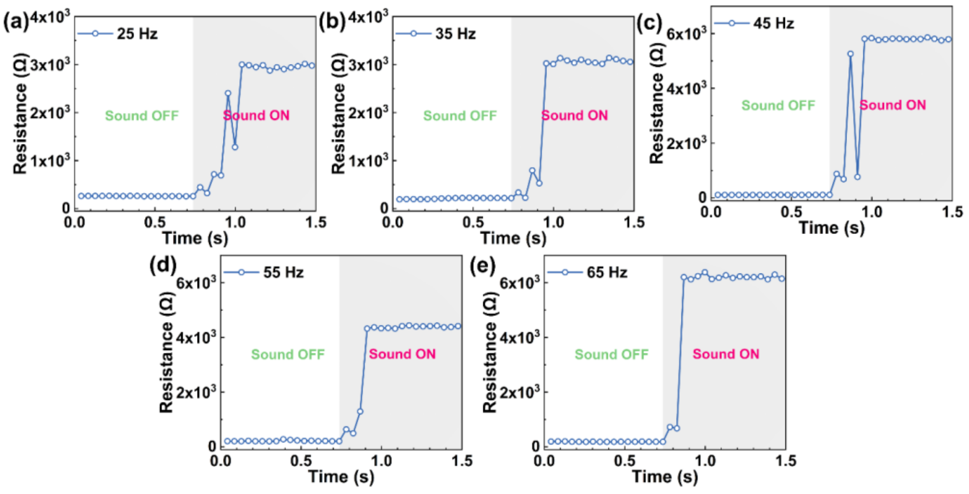

Figure S13. Evolution of the device resistance stimulated by different frequency sound waves. (a) 25 Hz. (b) 35 Hz. (c) 45 Hz. (d) 55 Hz. (e) 65 Hz.

Section 15. Cumulative probability plot of acoustic-HRSs induced by different frequencies.

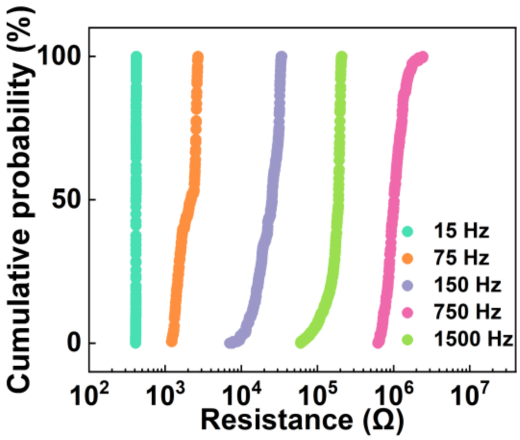

Figure S14. Cumulative probability plot of acoustic-HRSs induced by different frequencies.

Section 16. Cumulative probability plot of acoustic-HRSs induced by different SP.

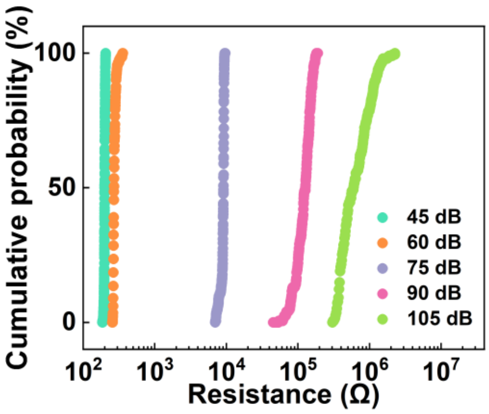

Figure S15. Cumulative probability plot of acoustic-HRSs induced by different SPL.

Section 17. Summary of performance parameters of other memristors and this work.

Table S2. Summary of performance parameters of other memristors and this work.

Structure	V_{set}/V_{reset} (V)	Switching Speed (ns)	ON/OFF Ratio	Endurance (Cycles)	Retention (s)	Ref
Au/NbO_x/NbS_2/MoS_2/Au	+1.20/−1.10	100	10	1.5×10^3	-	[8]
Au/Ti/*h*-BN/graphene	+5.20/−3.00	-	10^3	5×10^1	-	[9]
Pd/Cr/Si/graphene/Cr/Pd	+5.00/-	-	-	10^3	10^4	[10]
Au/NbO_x/NbS_2/MoS_2/Au	+1.20/−1.10	-	10	1.5×10^3	-	[11]
Au/ $(MA)_3Bi_2I_9$/ITO	+1.60/−0.60	-	10^2	3×10^2	10^4	[12]
NiO/BA_2PbBr_4/ZnO/FTO	+2.30/−3.00	-	10	2×10^2	-	[13]
Pt/$CsPbBr_3$/FTO	+1.60/−2.00	-	2.5×10^1	10^2	-	[14]
Ag/MoO_3/$MAPbI_3$/ITO	+0.25/−1.60	-	10^2	-	10^3	[15]
Al/$MAPbI_3$/p^+-Si	−3.15/+2.21	-	10^3	2×10^2	10^4	[16]
EGaIn/$MAPbI_3$/PEDOT:PSS/ITO	+0.56/−0.87	340 ns	Electrical:10^4 Acoustic: 10^3	5×10^3	5×10^4	This work

References

[1] Y. Zhang, Y. Liu, Z. Yang, S. F. Liu, *J. Energy Chem.* **2018**, 27, 722.

[2] J. Ding, L. Jing, X. Cheng, Y. Zhao, S. Du, X. Zhan, H. Cui, *J. Phys. Chem. Lett.* **2018**, 9, 216.

[3] F. Brivio, J. M. Frost, J. M. Skelton, A. J. Jackson, O. J. Weber, M. T. Weller, A. R. Goñi, A. M. A. Leguy, P. R. F. Barnes, A. Walsh, *Phys. Rev. B* **2015**, 92, 144308.

[4] M. Park, N. Kornienko, S. E. Reyes-Lillo, M. Lai, J. B. Neaton, P. Yang, R. A. Mathies, *Nano Lett.* **2017**, 17, 4151.

[5] V. H. Damle, L. Gouda, S. Tirosh, Y. R. Tischler, *ACS Appl. Energ. Mater.* **2018**, 1, 6707.

[6] C. Quarti, E. Mosconi, F. De Angelis, *Phys. Chem. Chem. Phys.* **2015**, 17, 9394.

[7] J. M. Frost, K. T. Butler, A. Walsh, *APL Mater.* **2014**, 2, 081506.

[8] B. Wang, H. Luo, X. Wang, E. Wang, Y. Sun, Y. Tsai, H. Zhu, P. Liu, K. Jiang, K. Liu, *ACS Nano* **2019**, 14, 175.

[9] H. Park, M. A. Mastro, M. J. Tadjer, J. Kim, *Adv. Electron. Mater.* **2019**, 5, 1900333.

[10] S. Choi, J. Choi, J. C. Kim, H. Y. Jeong, J. Shin, S. Jang, S. Ham, N. D. Kim, G. Wang, *Nano Energy* **2021**, 84, 105947.

[11] B. Wang, H. Luo, X. Wang, E. Wang, Y. Sun, Y. Tsai, H. Zhu, P. Liu, K. Jiang, K. Liu, *ACS Nano* **2019**, 14, 175.

[12] B. Hwang, J. Lee, *Nanoscale* **2018**, 10, 8578.

[13] M. Kumar, H. Kim, D. Y. Park, M. S. Jeong, J. Kim, *Nanoscale* **2018**, 10, 11392.

[14] H. Liu, Y. Wu, Y. Hu, *Ceram. Int.* **2017**, 43, 7020.

[15] Z. Xu, Z. Liu, Y. Huang, G. Zheng, Q. Chen, H. Zhou, *J. Mater. Chem. C* **2017**, 5, 5810.

[16] D. J. Kim, Y. J. Tak, W. G. Kim, J. K. Kim, J. H. Kim, H. J. Kim, *Adv. Mater.*

Interfaces **2017**, 4, 1601035.
